# Rolling the evolutionary dice: *Neisseria* commensals as proxies for elucidating the underpinnings of antibiotic resistance mechanisms and evolution in human pathogens

**DOI:** 10.1128/spectrum.03507-23

**Published:** 2024-01-05

**Authors:** Kelly M. Frost, Sierra L. Charron-Smith, Terence C. Cotsonas, Daniel C. Dimartino, Rachel C. Eisenhart, Eric T. Everingham, Elle C. Holland, Kainat Imtiaz, Cory J. Kornowicz, Lydia E. Lenhard, Liz H. Lynch, Nadia P. Moore, Kavya Phadke, Makayla L. Reed, Samantha R. Smith, Liza L. Ward, Crista B. Wadsworth

**Affiliations:** 1 Rochester Institute of Technology, Thomas H. Gosnell School of Life Sciences, Rochester, New York, USA; Griffith University - Gold Coast Campus, Southport, Gold Coast, Australia

**Keywords:** experimental evolution, *Neisseria*, azithromycin, penicillin, antibiotic resistance, experiential learning

## Abstract

Species within the genus *Neisseria* are adept at sharing adaptive allelic variation, with commensal species repeatedly transferring resistance to their pathogenic relative *Neisseria gonorrhoeae*. However, resistance in commensals is infrequently characterized, limiting our ability to predict novel and potentially transferable resistance mechanisms that ultimately may become important clinically. Unique evolutionary starting places of each *Neisseria* species will have distinct genomic backgrounds, which may ultimately control the fate of evolving populations in response to selection as epistatic and additive interactions coerce lineages along divergent evolutionary trajectories. Alternatively, similar genetic content present across species due to shared ancestry may constrain existing adaptive solutions. Thus, identifying the paths to resistance across commensals may aid in characterizing the *Neisseria* resistome—or the reservoir of alleles within the genus as well as its depth. Here, we use *in vitro* evolution of four commensal species to investigate the potential and repeatability of resistance evolution to two antimicrobials, the macrolide azithromycin and the β-lactam penicillin. After 20 days of selection, commensals evolved resistance to penicillin and azithromycin in 11/16 and 12/16 cases, respectively. Almost all cases of resistance emergence converged on mutations within ribosomal components or the *mtrRCDE* efflux pump for azithromycin-based selection and *mtrRCDE*, *penA*, and *rpoB* for penicillin selection, thus supporting constrained adaptive solutions despite divergent evolutionary starting points across the genus for these particular drugs. Though drug-selected loci were limited, we do identify novel resistance-imparting mutations. Continuing to explore paths to resistance across different experimental conditions and genomic backgrounds, which could shunt evolution down alternative evolutionary trajectories, will ultimately flesh out the full *Neisseria* resistome.

**IMPORTANCE:**

*Neisseria gonorrhoeae* is a global threat to public health due to its rapid acquisition of antibiotic resistance to all first-line treatments. Recent work has documented that alleles acquired from close commensal relatives have played a large role in the emergence of resistance to macrolides and beta-lactams within gonococcal populations. However, commensals have been relatively underexplored for the resistance genotypes they may harbor. This leaves a gap in our understanding of resistance that could be rapidly acquired by the gonococcus through a known highway of horizontal gene exchange. Here, we characterize resistance mechanisms that can emerge in commensal *Neisseria* populations via *in vitro* selection to multiple antimicrobials and begin to define the number of paths to resistance. This study, and other similar works, may ultimately aid both surveillance efforts and clinical diagnostic development by nominating novel and conserved resistance mechanisms that may be at risk of rapid dissemination to pathogen populations.

## INTRODUCTION

The emergence of antibiotic resistance within bacterial populations is mediated by natural selection, whereby mutations encoding drug-protective mechanisms are produced stochastically and subsequently increase in frequency as a result of only the cells harboring these mutations surviving exposure events. However, a key question for both understanding the evolutionary process and also the enhancement of surveillance efforts is: how repeatable and predictable is resistance evolution at the genotypic level? Two alternate hypotheses can be advanced: (i) adaptive landscapes are constrained to one or few solutions (i.e., genotypic constraint) or (ii) a multitude of fitness peaks exist created by many mutations imparting similar phenotypic outcomes. Many prior studies support some level of genotypic constraint on resistance evolution at the strain or species level (1–5); however, less frequently has the repeatability of resistance evolution been interrogated across species’ boundaries. Applying selection across different genomic backgrounds at the species level may lead us to predict a higher likelihood of divergent evolutionary outcomes, with different mutations giving rise to similar phenotypic resistance in different species. We may predict this given that the pre-existing suite of potentially additive and/or epistatically interacting mutations already present in each species’ genomes will likely be unique as a result of both genetic drift since the time of lineage divergence and also niche-specific adaptation. However, if genotypic convergence is observed across species, this suggests constrained ranges of adaptive solutions between high-level taxonomic groupings (e.g., genera, families, etc.) due to their shared ancestral history and conserved genetic makeup. Here, we begin to interrogate this question: does genotypic constraint or divergence govern the emergence of resistance evolution within the genus *Neisseria*?

The genus *Neisseria* is composed of several Gram-negative, typically diplococcoid, oxidase-positive, and often catalase-positive species, which most frequently colonize the nasopharyngeal or oral niche in humans or animals (6). There are, at a minimum, 10 human-associated species that likely evolved from a common ancestor that colonized an early humanoid (7). Interestingly, contemporary *Neisseria* has distinct colonization sites within the oro- and nasopharynx suggesting ecological and physiological divergence between species since that initial colonization event (8). Some species are carried harmlessly as commensals in 100% of healthy human adults and children (*Neisseria cinerea*, *Neisseria polysaccharea*, *Neisseria lactamica*, *Neisseria mucosa*, *Neisseria oralis*, *Neisseria subflava*, *Neisseria elongata* [atypical rod], and *Neisseria bacilliformis* [atypical rod]); however, two species have significant pathogenic potential (*Neisseria gonorrhoeae* and *Neisseria meningitidis*) and are carried in a smaller percentage of the population (between 0.01% and 10%) (9–13). *N. gonorrhoeae* is unique within the genus in that in addition to colonizing the nasopharyngeal mucosa, it also routinely colonizes the urogenital tract and rectum and causes the sexually transmitted infection gonorrhea (14).

Within the *N. gonorrhoeae* population, rates of resistance to multiple classes of antimicrobials are rising. For example, according to the latest Gonococcal Isolate Surveillance Project (GISP) report (15), ~15% of surveyed isolates were resistant to penicillin, ~20% resistant to tetracycline, 33.2% to ciprofloxacin, 5.8% to azithromycin, and 0.3% to cefixime in the United States; although resistance (≥0.25 µg/mL) was not observed in 2020 to ceftriaxone, isolates with reduced susceptibly have been identified in previous years (2017–2019) as part of the GISP collection (15). Additionally, surveillance studies in other countries have identified higher rates of circulating ceftriaxone resistance (e.g., 4.2% in Taiwan [16] and 16% in Guangdong, China [17]) with recent observations indicating global dissemination (Japan, China, Europe, Australia, North America, and Southeast Asia) of high-level ceftriaxone-resistant strains (18–23).

Though the genetic basis of some resistance phenotypes appears to be exclusively encoded by recurrently acquired mutations {i.e., ciprofloxacin resistance is almost always caused by amino acid substitutions in the DNA gyrase subunit A (GyrA S91F and D95G/D95A [24, 25])}, the complete genetic bases of other resistance phenotypes are currently not fully described and/or imparted by additive or epistatically interacting loci for which the combined effects must be quantified (e.g., penicillin [26–30] and azithromycin [25, 31] resistance). For example, known mutations decreasing susceptibility to penicillin have been described in: *penA* (encoding penicillin-binding protein 2 [PBP2]) (32), *ponA* (penicillin-binding protein 1 [PBP1] (28), Mtr efflux pump components (29), the *major outer membrane porin protein P1B* allele (26), and β-lactamase harboring plasmids (33); however, at least one unknown untransformable resistance determinant exists (“factor X”) (34). In addition, most of the described azithromycin resistance can be gained through mutations in Mtr efflux pump components (35), mutations in the 23S rRNA azithromycin-binding sites (C2611T and A2059G) (36, 37), and ribosomal protein mutations (38). However, a large proportion of lower-level resistance remains unexplained (25, 38). Thus, experimentally interrogating the paths to resistance and their repeatability in *Neisseria* will become an important step for identifying novel contributing mutations, identifying the combinations of loci that contribute to polygenic resistance, and understanding their potential prevalence and evolution within populations.

Studies on the paths to resistance within gonococci have previously been explored *in vitro* (39–44). However, gonococci, in addition to gaining resistance through *de novo* mutations, are also superbly adept at acquiring resistance from their close commensal relatives (5, 31, 45–47). This allelic exchange across *Neisseria* species likely occurs in their shared colonization sites of the naso- and oropharyngeal niches (8), with the whole genus often being referred to as a consortium with “fuzzy” borders due to the high frequency of DNA donation through horizontal gene transfer (48–50). Commensal species thus serve as a bubbling cauldron of new adaptive solutions and reservoirs of resistance for gonococci, with each species containing a unique genomic background in which novel resistance genotypes may emerge. Therefore, expanding the investigation on the repeatability of evolution to the entire genus may serve two important goals in the fight against the spread of resistance in gonococci: (i) identifying resistance phenotypes for which a multitude of genotypic paths exist, either within distinct genomic contexts or across several and (ii) determining which drugs and/or drug classes have limited adaptive solutions within the genus. Both of these findings may guide the development of nucleic acid-based resistance tests [i.e., nucleic acid-based resistance tests (NAAT) or whole genome sequencing (WGS)] for surveillance programs by defining the scope of mutations that must be surveyed.

Here, we begin to interrogate the paths to resistance to two drugs with as-of-yet not fully identified genotypic bases within the pathogenic *Neisseria*. We use four different genomic contexts across the *Neisseria* genus (*N. cinerea*, *N. subflava*, *N. elongata*, and *Neisseria canis*) and select for increasing minimum inhibitory concentrations (MICs) by passing each species across selective gradients as previously described (5). Though the scope of this initial and a prior study (5) has been limited (i.e., limited species and experimental replicates), we imagine that by continuing to “roll the evolutionary dice” we will ultimately coalesce on the possible paths to resistance and their quantity, addressing the repeatability of evolution to different drug classes across the genus. Finally, both this and our previous study (5) were conducted as part of exercises within undergraduate classrooms at the Rochester Institute of Technology, highlighting the power of experimental evolution in addressing fundamental questions impacting global public health, while also providing important experiential learning opportunities for entry-level students.

## RESULTS

### Rolling the dice: evolving *Neisseria* commensals

Four *Neisseria* commensal species were selected as distinct evolutionary starting points for antibiotic selection (*N. cinerea* [AR-0944], *N. subflava* [AR-0953 and AR-0957], *N. elongata* [AR-0945], and *N. canis* [AR-0948]). All are human-associated commensals except for *N. canis*, which colonizes the oral cavity of dogs and cats but has also been isolated from human patients with dog and cat bite wounds (51–53). All isolates had been phenotyped for their MICs to penicillin and azithromycin (Table 1), and the majority were sequenced previously (54). One isolate, AR-0944, was sequenced as a part of this study (accession: JAXUDV000000000; length 2.13 Mbp, 131 contigs, N50 = 250 kbp, GC content 50.78%).

**TABLE 1:** MICs for ancestral strains and average MICs for evolved strains

Ancestral strains	Azi MIC (μg/mL)	Average azi MIC (μg/mL) evolved (*n* = 4)	Pen MIC (μg/mL)	Average pen MIC (μg/mL) evolved (*n* = 4)
AR-0944 (*N. cinerea*)	8	152	0.38	12
AR-0945 (*N. elongata*)	0.5	0.69	0.25	6.72
AR-0948 (*N. canis*)	0.38	64	0.25	5.44
AR-0953 (*N. subflava*)	2	224	1.5	†
AR-0957 (*N. subflava*)	8	†^*a*^	1	3.69

^*a*^ † AR-0953 was only selected with azithromycin, and AR-0957 was only selected with penicillin; see discussion for further details.

**Fig 1:**
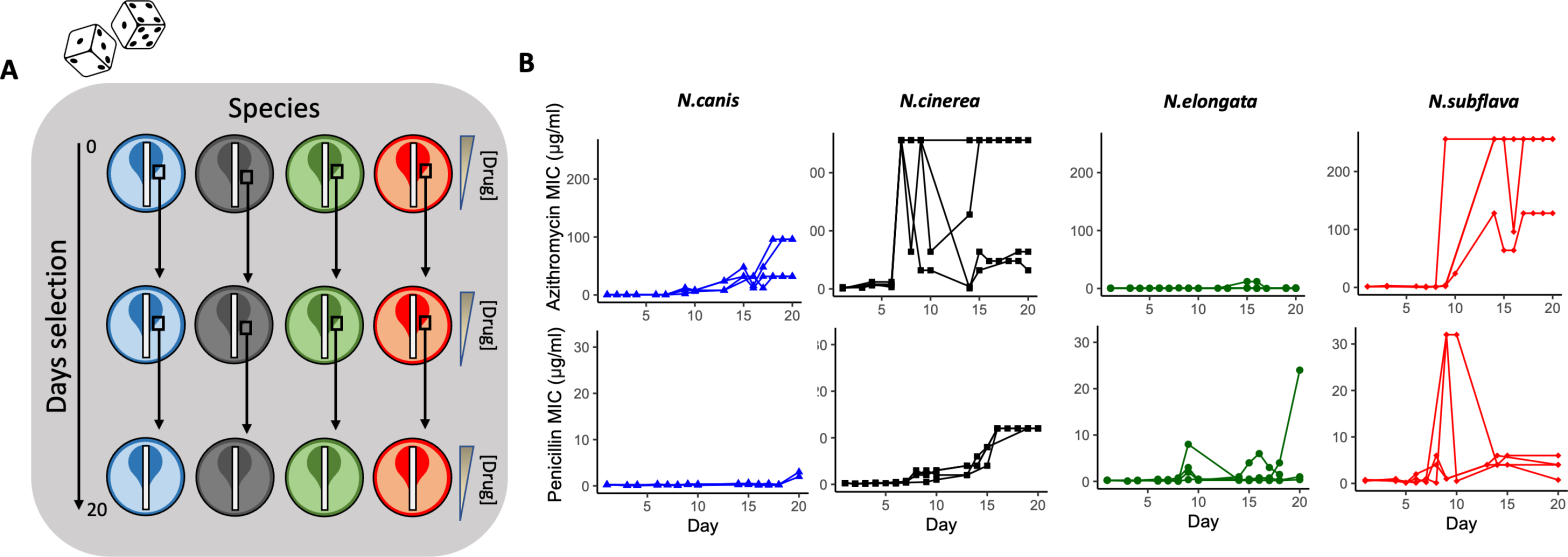
Azithromycin and penicillin-mediated selection of four species of commensal *Neisseria*. (**A**) Four species with distinct genetic backgrounds were selected as unique starting points for *in vitro* evolution to two antimicrobials. Each experimental replicate and species/drug combination can be envisioned as an independent “roll of the dice,” in which new derived mutations and evolutionary trajectories may emerge. In brief, four experimental replicates were passaged for each species and drug combination for 20 days on selective gradients created with Etest strips. Cells for each passage were selected by sweeping the entire ZOI and a 1 cm band in the bacterial lawn surrounding the ZOI. (**B**) Overall, after 20 days, evolved azithromycin MICs tended to be higher than of penicillin MICs, with species also differing in their evolutionary trajectories toward elevated MICs within a drug class.

For each species and drug combination, four replicate lineages were passaged with selection created by application of Etest strips on standard growth media as previously described (5; Fig. 1). Cells were passaged for 20 days by sweeping the entire zone of inhibition (ZOI) and a 1 cm band surrounding the ZOI and plating any collected cells on new selective growth media. For azithromycin, the average MICs of evolved *N. cinerea* (MIC = 152 ± 120.79 µg/mL), *N. canis* (64 ± 36.95 µg/mL), and *N. subflava* (224 ± 64 µg/mL) lineages crossed the breakpoint of reduced susceptibility as defined by the Clinical and Laboratory Standards Institute (CLSI) guidelines for *N. gonorrhoeae* of ≥2 µg/mL (55). *N. elongata* lineages, however, did not surpass this breakpoint (0.69 ± 0.36 µg/mL). For penicillin, the average MICs for evolved lineages of all species surpassed the CLSI-defined breakpoint concentration of ≥2 µg/mL (55): *N. cinerea* (MIC = 12 ± 0 µg/mL), *N. elongata* (6.75 ± 11.53 µg/mL), *N. canis* (5.44 ± 1.38 µg/mL), and *N. subflava* (3.69 ± 2.17 µg/mL). Control populations (*n* = 3 per species) with no drug selection showed no significant increase in azithromycin or penicillin MICs compared to the ancestral stocks (Table S1).

**Fig 2:**
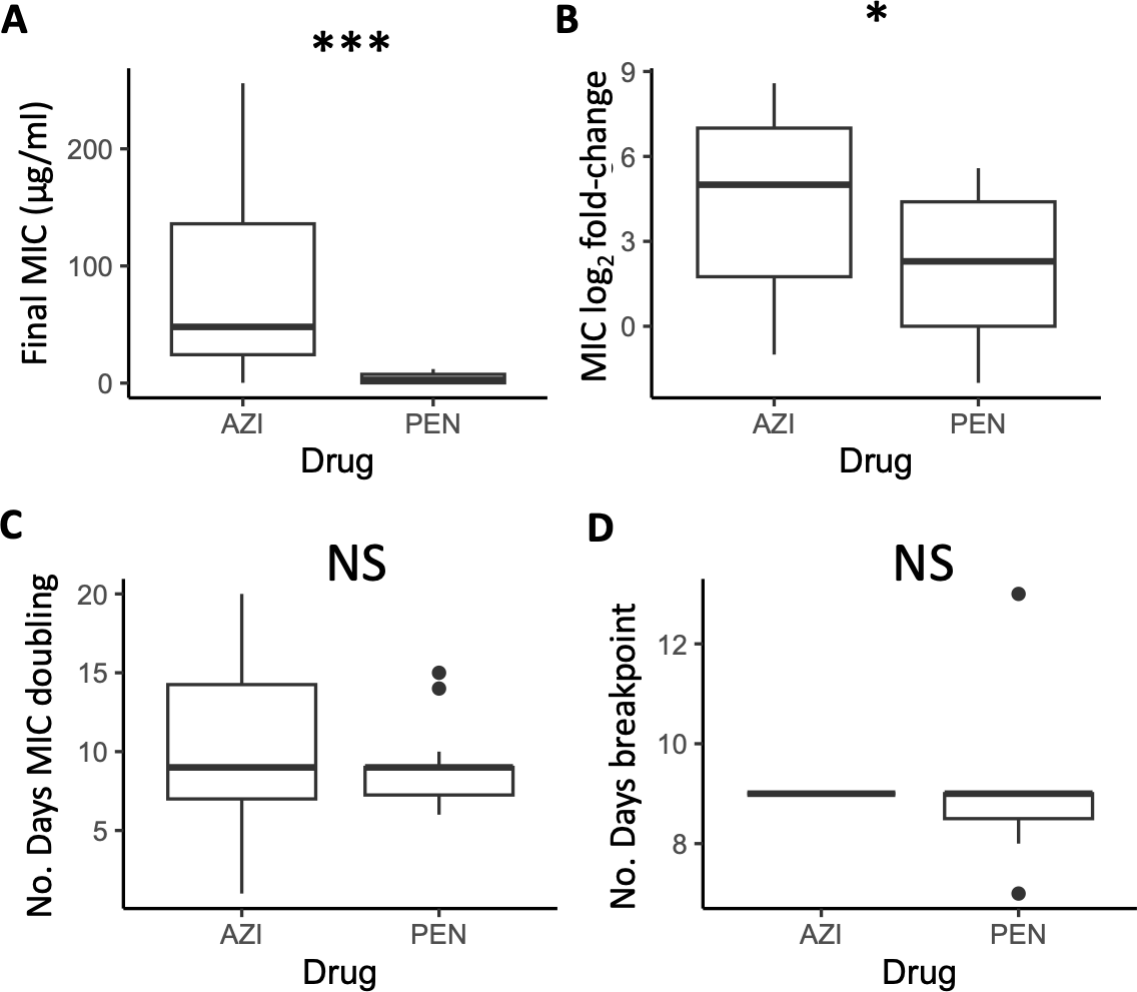
Across all species, evolved azithromycin MICs were significantly elevated compared to penicillin MICs in both (**A**) their final values (*P* < 0.0001) and (**B**) their fold-increase from ancestral MICs (*P* < 0.01). (**C**) The time for MICs to double was not significantly different between drugs (*P* > 0.05), as was the number of days to surpass the breakpoint value as defined by CLSI guidelines for *N. gonorrhoeae* (*P* > 0.05).

**Fig 3:**
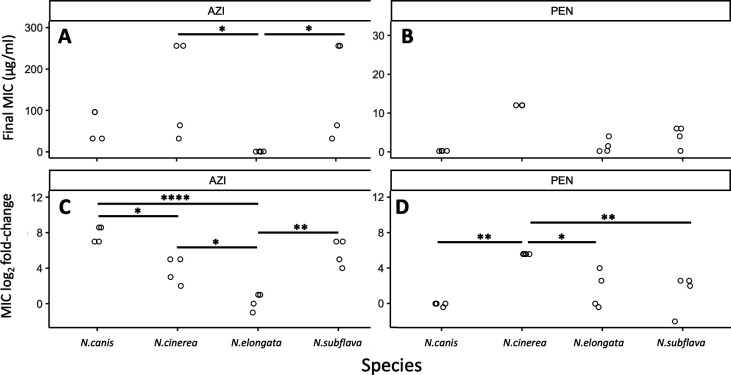
Evolved MICs and MIC log-fold change values separated by drug and species. (**A**) For azithromycin, *N. subflava* and *N. cinerea* had significantly higher MICs compared to *N. elongata* after selection (Tukey’s HSD: *P* = 0.036 and *P* = 0.036, respectively). (**B**) Species were not significantly different between any contrast for penicillin. However, between species fold-change in MIC after evolution was significantly different for (**C**) four contrasts for azithromycin (Tukey’s HSD: *P* < 0.05) and (**D**) three contrasts for penicillin (Tukey’s HSD: *P* < 0.01).

Final recorded MICs for azithromycin (92.17 ± 25.57 µg/mL) were significantly higher across all commensal species compared to the MICs for penicillin (4.45 ± 1.23 µg/mL; *W* = 38.5, *P* = 0.00073; Fig. 2A). Azithromycin MIC fold-changes (4.39 ± 0.77) were also significantly higher than that of penicillin MICs (2.08 ± 0.65) across species (*W* = 74, *P* = 0.043; Fig. 2B). The number of days for MICs to double for azithromycin (10.75 ± 1.34) compared to penicillin (9.07 ± 0.70) were not significantly different (*W* = 92.5, *P* = 0.41; Fig. 2C) nor was the day the CLSI resistance breakpoint was passed at 9.0 ± 0 and 9.0 ± 0.45, respectively (*W* = 18, *P* value = 0.56; Fig. 2D), with species starting with above breakpoint values at the beginning of the experiment omitted for this last analysis. Between species for azithromycin, *N. subflava* and *N. cinerea* had significantly higher evolved MICs compared to *N. elongata* (Tukey’s HSD: *P* = 0.036 and *P* = 0.036, respectively; see also Fig. 3A; Table S1). There were no significant differences for final MICs between species for penicillin (Fig. 3B). However, between species fold-change in MIC was significantly different for four contrasts for azithromycin (Tukey’s HSD: *P* < 0.05; Fig. 3C) and three contrasts for penicillin (Tukey’s HSD: *P* < 0.01; Fig. 3D).

### The frequency and identity of derived mutations

**Fig 4:**
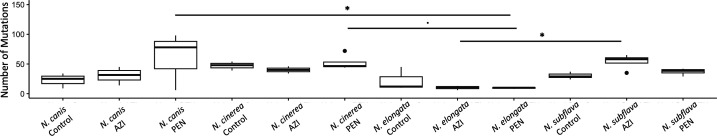
The number of derived mutations after the 20-day long experiment for azithromycin and penicillin selected lines as well as control lineages. For penicillin, both *N. canis* and *N. cinerea* had significantly more derived mutations compared to *N. elongata* (Tukey’s HSD: *P =* 0.02 *and P* = 0.059, respectively), and for azithromycin, *N. subflava* had significantly more derived mutations compared to *N. elongata* (Tukey’s HSD: *P =* 0.045).

For each evolved lineage, a single colony was picked for further characterization and whole-genome sequencing (Table S1). There were no significant differences between the number of derived mutations after the 20-day long experiment between drugs across all species; however, each species and interaction between drugs and species (two-way ANOVA: *P* = 0.0008) had a significant and nearly significant (two-way ANOVA: *P* = 0.055) impact on the number of derived mutations, respectively. *N. elongata* had significantly fewer derived mutations compared to *N. canis* (Tukey’s HSD: *P* = 0.02), *N*. cinerea (Tukey’s HSD: *P =* 0.0007), and *N. subflava* (Tukey’s HSD: *P* = 0.004). Differences in the number of derived mutations did not appear to be correlated to growth rate as doubling times were calculated at 224 minutes for *N. cinerea*, 258 minutes for *N. elongata*, 299 minutes for *N. canis*, and 715 minutes for *N. subflava* (Fig. S1), which may support different baseline mutation rates between species. While *Neisseria* pathogens are reported to divide more rapidly (40–60 minutes), longer division times have been reported for commensals previously (56). When separated by drug class, for penicillin, both *N. canis* and *N. cinerea* had significantly more derived mutations compared to *N. elongata* (Tukey’s HSD: *P =* 0.02 *and P* = 0.059 respectively; Fig. 4), and for azithromycin, *N. subflava* had significantly more novel mutations compared to *N. elongata* (Tukey’s HSD: *P =* 0.045; Fig. 4).

**Fig 5:**
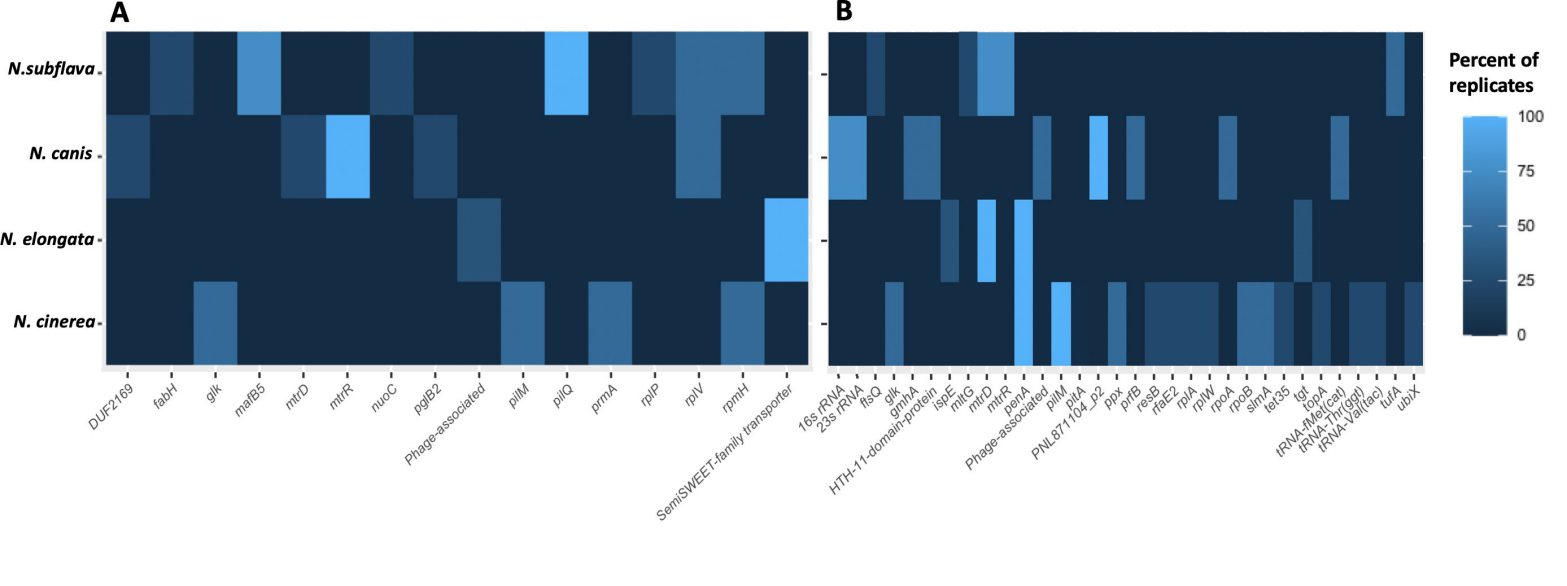
Identity of derived mutations in CDSs for drug-selected lineages for (**A**) azithromycin and (**B**) penicillin. The percentage of reoccurring mutations within a given gene is displayed as a heatmap, with brighter blue coloration indicating the more frequent occurrence of a mutation within a CDS in replicate evolved lineages for each species.

Mutations within coding domain sequences (CDSs) and rRNA sequences were identified for all evolved lineages and, after correcting for mutations also present in control lineages with no drug exposure, were considered candidates for imparting resistance (Fig. 5; Table 2). For azithromycin, all replicate lineages of *N. subflava*, *N. canis*, and *N. cinerea* evolved resistance; however, none of the *N. elongata* strains did (Fig. 1). The most frequent mutation occurring in *N. subflava* lineages was located within *pilM*, a component of type IV pili (Tfp). Additional mutations that emerged included those in beta-ketoacyl-ACP synthase III (encoded by *fabH)*, a Maf-family transcription factor (*mafB5)*, NADH-quinone-oxidoreductase (*nqr)*, the 50S ribosomal protein L16 (*rplP)*, the 50S ribosomal protein L22 (*rplV)*, and the 50S ribosomal L34 protein *(rpmH)*. For *N.canis*, the most frequent mutations occurred in the repressor of the Mtr efflux pump (*mtrR),* followed by *rplV*, *duf2169*, the inner membrane component of the Mtr efflux pump (*mtrD)*, and a component of the glycan biosynthesis pathway (*pglB2)*. Finally, for *N. cinerea*, mutations emerged in glucokinase (*glk)*, ribosomal protein L11 methyltransferase *(prmA)*, and *rpmH*. For penicillin, all replicate evolved lineages gained resistance except for one *N. elongata* strain and all *N.subflava* strains; however, each of these lineages developed increased MICs compared to the ancestral strains and had MICs ≥ 1 µg/mL. Mutations in *N. subflava* lineages that emerged include those in *mtrD* and *mtrR*, elongation factor Tu *(tufA)*, and the endolytic murein transglycosylase *(mltG)*. The most frequent mutations in *N. canis* include those in the 16S and 23S rRNAs, followed by those in *PNL71104_P2*, phosphoheptose isomerase *(gmhA)*, an HTH11-domain coding protein, a phage-associated protein, peptide chain release factor 2 (*prfB*), DNA-directed RNA polymerase subunit alpha (*rpoA*), and *tRNA-fMet(cat*). In *N. elongata*, derived mutations include those in *mtrD*, PBP2 (*penA*), 4-diphosphocytidyl-2-C-methyl-D-erythritol kinase *(ispE)*, and queuine tRNA-ribosyltransferase *(tgt)*. Finally in *N. cinerea*, mutations included those in *penA*, *pilM*, *glk*, phosphate transporter *(pitA)*, exopolyphosphatase *(ppx)*, DNA-directed RNA polymerase subunit beta *(rpoB)*, and HTH-type protein slmA *(slmA)*, along with some additional singleton mutations (Fig. 5).

**TABLE 2:** Loci and mutations impacting resistance to azithromycin and penicillin in *N. Gonorrhoeae* and the identities of derived variants within those loci uncovered in this study^*a*^^,^^*b*^

				Emergence in commensals and derived variants in locus			
Antibiotic	Loci contributing to resistance to *N. gonorrhoeae*	Specific mutations in *N. gonorrhoeae*	References	*N. canis*	*N. cinerea*	*N. elongata*	*N.subflava*
Azithromycin	*23S* rRNA	A2059G and C2611T	(36, 57)	–^*c*^	–	–	–
	*mtrR*	A39T and G45D	(58, 59)	A37V, G172D, and premature stop	–	–	–
	*mtrR-CDE* promoter	*A-56C, −57delA, G-131A, G-120A substitution,* and *mtr120*	(31, 58, 60)	–	–	–	–
	*mtrD*	E823K	(31)	E823K	–	–	–
	*rplV*	Tandem duplications (83-KGPSLK and 90-ARAK)	(25)	91-KRFQARAKG and 91-GRGNRIQARAKG	–	–	78-KGPSLKRFQARA
	*rplD*	G70D	(38)	–	–	–	–
	*rpmH*	7-PSVTKRKR, 7-PSVTNTYQP, and 7-LKRTYQ	(40)	–	6-HHETHLS and 6-QLMKRTYQ	–	8-SVKRTYQP
	*macAB* promoter	G48T	(46)	–	–	–	–
	*ermA, ermB, ermC, ermF* genes	Absence > presence	(61, 62)	–	–	–	–
	*ereA, ereB* genes	Absence > presence	(63)	–	–	–	–
Penicillin	*blaTEM*	Absence > presence	(64)	–	–	–	–
	*mtrR*	A39T and G45D	(58, 59)	–	–	–	T11I
	*mtrD*	ND	–	–	–	V139G, F604I, and A1009T	L996I
	*mtrR-CDE* promoter	*A-56C, −57delA, G-131A, G-120A substitution,* and *mtr120*	(31, 58, 60)	–	–	–	–
	*penA*	I312M, V316P/T, ins346D, T483S, A501P/T/V, G542S, G545S, and P551S	(65–67)	–	F518S, V548E, and A549E	P399S, V574E, and A581S	–
	*ponA*	L421P	(28)	–	–	–	–
	*porB1b*	G120K, A121N/D	(27)	–	–	–	–
	*rpoB*	ND	(68)	–	E345A, P591S	–	–

^*a*^ NFC = Not fully characterized.

^*b*^ ND = Not described.

^*c*^ ”-” No mutations observed.

## DISCUSSION

Commensal *Neisseria* has repeatedly donated resistance alleles to their pathogenic relative *N. gonorrhoeae* (31, 45–47) and beyond doubt serve as a bubbling cauldron of new adaptive solutions to address “the antibiotic crisis” that *N. gonorrhoeae* faces. However, we do not yet understand the full suite of resistance alleles that commensal *Neisseria* can carry, if the pool of mechanisms is large or small and if the pool size varies by antibiotic. Here, we roll the evolutionary dice using antibiotic selection across divergent commensal *Neisseria* genomic contexts to begin to answer three important questions: (i) what are the identities of resistance mutations that emerge in commensals in response to selection, (ii) are the paths to resistance constrained or diverse, and (iii) do resistance mutation identities and paths vary by drug class?

Azithromycin is a macrolide antibiotic that inhibits protein synthesis by binding to the 23S rRNA component of the 50S ribosome. Mutations that impact the conformation or block the binding site of the drug have previously been described in *N. gonorrhoeae* to impart resistance and include mutations in the 23S rRNA azithromycin-binding sites (C2611T and A2059G) (36, 57), a G70D mutation in the RplD 50S ribosomal protein L4^38^, and *rplV* tandem duplications (25). Here, we find a suite of variants that emerged post-selection within the CDSs encoding ribosomal proteins. For example, in both *N. subflava* and *N. canis*, we uncovered mutations emerging in *rplV* encoding the 50S ribosomal protein L22, with 2/4 *N*. *subflava* lineages (78-KGPSLKRFQARA) and 2/4 *N. canis* (91-KRFQARAKG and 91-GRGNRIQARAKG) lineages evolving tandem duplications or insertions within this gene. Similar duplications in this region have previously been predicted to block the azithromycin-binding site in *N. gonorrhoeae* (25). In-frame insertions in *rpmH*, which encodes the 50S ribosomal L34 protein, were also frequent and found within 2/2 surviving *N. cinerea* and 2/4 *N. subflava* strains. *N. cinerea* strains both evolved distinct *rpmH* variants (6-HHETHLS and 6-QLMKRTYQ), while *N. subflava* strains evolved the same variant (8-SVKRTYQP). Similar duplications in *rpmH* have been previously described in *N. elongata* (5) and *N. gonorrhoeae* (40) and have been found to be casual to high-level azithromycin resistance through a transformation in *N. elongata* (5). Thus, these are the likely mutations imparting high-level resistance in *N. cinerea* strains within this study. Interestingly, the *N. elongata* strains evolved in this study did not evolve reduced azithromycin susceptibility (Fig. 1; Table 1); however, in our prior work (5), only 44% of replicate *N. elongata* lineages evolved resistance, and only 43% of these resistant isolates gained resistance through mutations in *rpmH.* With only four replicate *N. elongata* strains selected in this study, we speculate that we did not have sufficient power to uncover *rpmH* variants in *N. elongata*, as we have previously found they have a slower growth rates (5) and, therefore, may have been outcompeted. Finally, we find evidence for a duplication within the *rplP* gene encoding the 50S ribosomal protein L16 within a single *N. subflava* strain; however, we find no difference in MICs between this strain which also harbors a *rplV* duplication and a second strain with just a *rplV* duplication, suggesting that the variant uncovered in *rplP* may not contribute to the elevated MICs observed. Manoharan-Basil et al. (2021) (69) describe multiple recombination events in genes encoding ribosomal proteins across pathogenic and commensal *Neisseria*, supporting the possibility of transfer of these types of resistance mutations in natural *Neisseria* populations.

The multiple transferable resistance efflux pump (Mtr) is a primary mechanism by which *N. gonorrhoeae* gains resistance to both azithromycin and penicillin. The Mtr efflux pump is composed of the MtrC-MtrD-MtrE cell envelope proteins, which together export diverse hydrophobic antimicrobial agents, such as antibiotics, nonionic detergents, antibacterial peptides, bile salts, and gonadal steroidal hormones from the cell (70–73). Overexpression of the pump, through mutations that ablate or decrease the expression of the repressor of the pump (MtrR), has been demonstrated to increase resistance to both azithromycin and penicillin (25, 29, 74, 75), and substitutions within the inner membrane component MtrD have been shown to decrease susceptibility to azithromycin (31, 46). Here, in response to azithromycin-based selection, all four experimental replicates of *N. canis* evolved mutations in MtrR: two with a G172D substitution, one A37V, and one insertion impacting the reading frame and resulting in a premature stop codon. Then, 3/4 replicates of *N. subflava* evolved *mtrR* mutations in response to penicillin exposure which resulted in a T11I substitution in MtrR. MtrR mutations uncovered in commensals within the study have not been described in *N. gonorrhoeae* to impart azithromycin-reduced susceptibility previously; however, the A37V mutation in *N. canis* is proximal to the predicted helix-turn-helix domain of the MtrR protein known to be important in DNA-binding activity in *N. gonorrhoeae* (59). MtrD mutations also emerged in response to penicillin-selection in *N. subflava* (L996I) and *N. elongata* (with all three strains carrying different mutations: V139G, F604I, or A1009T). Finally, a mutation encoding MtrD E823K also emerged in 1/4 *N. canis* strains after azithromycin selection. Interestingly, this last E823K MtrD substitution was predicted to be the causal mutation in mosaic commensal *Neisseria* alleles imparting azithromycin resistance and transferred to *N. gonorrhoeae* (31, 46).

β-lactams, such as penicillin, target the penicillin-binding proteins and inhibit cell wall biosynthesis. Mutations in PBP2 (encoded by *penA*) in particular have been well documented to impart elevated penicillin MICs in *N. gonorrhoeae* (28, 76) and also other β-lactams including the extended spectrum cephalosporin ceftriaxone, through both native gonococcal alleles (77) and non-native alleles acquired from commensal *Neisseria* (25, 47, 76, 78). These mutations act by lowering the affinity of the beta-lactam antibiotics for PBP2 and also by restricting the motions of PBP2 which are important for acylation by beta-lactams (79). Therefore, unsurprisingly, we observed multiple mutations emerge in *penA*, though only in two species: *N. elongata* and *N. cinerea*. Then, 3/3 surviving *N. elongata* evolved lines had *penA* mutations emerge: P399S, V574E, and A581S; all four experimental *N. cinerea* replicates evolved *penA* mutations encoding the amino acid substitutions: F518S, V548E, and A549E. All uncovered PenA mutations emerged in the transpeptidase domain of the protein (80). Mutations in another penicillin-binding protein, PBP1 encoded by *ponA*, also contribute to reduced penicillin susceptibility in gonococci (28); however, we did not observe the emergence of any *ponA* mutations within the commensal *Neisseria* evolved within this study.

Additional derived mutations of note that emerged after selection include those in the RNA polymerase and components of the pilus. Here, after penicillin selection, a *rpoA* mutation emerged in *N. canis*, and *rpoB* mutations emerged in *N. cinerea*. In *N. gonorrhoeae*, both RpoD (E98K and Δ92) and RpoB (R201H) mutations impact ceftriaxone susceptibility, another β-lactam antibiotic, likely through increased expression of PBP1 and reduced expression of D,D-carboxypeptidase (68). Here, the *rpoA* G147A nucleotide substitution in *N. canis* resulted in a silent change, so it does not likely contribute to elevated penicillin MICs; however, evolved *rpoB* mutations encoded amino acid substitutions (E345A and P591S) in 2/4 *N. cinerea* replicate lineages. Though these particular *rpoB* mutations have not been demonstrated to impart resistance in *Neisseria* previously, associations between mutations within this locus and ceftriaxone-reduced susceptibility in *N. gonorrhoeae* (68) *s*uggest that it may be important to further explore the relationship between new *rpoB* variants and resistance to β-lactam antibiotics. Another locus with a derived mutation that may reduce susceptibility to β-lactams is the lytic transglycosylase *mltG.* MltG acts as a terminase, ceasing peptidoglycan strand growth through cleavage of the growing glycan, and its deletion has been shown to increase susceptibility to penicillin in *N. gonorrhoeae* (81). In this study, a mutation emerged in a single *N. subflava* lineage in response to penicillin selection which resulted in a P293L substitution. Though rare, it may be an interesting candidate to further investigate with its known involvement in cell wall biosynthesis processes; however, its presence did not raise base MIC values from the ancestral strain, suggesting it has a marginal impact on resistance (if any). Finally, the pilus-associated mutations in PilM in *N. cinerea* in response to penicillin selection (4/4 isolates; 2 with a 1-bp deletion at position 404, and 2 with a 1-bp deletion at position 162) and azithromycin selection (1/4 isolates; 1 bp deletion at position 404), and PilQ in *N. subflava* (a 1 bp deletion at position 593 in 4/4 strains) in response to azithromycin all shift the reading frames resulting in non-function proteins. These mutations likely impact drug diffusion across the outer membrane and into the periplasm in some way similar to gonococci (82) (i.e., modification of the structure or assembly of the multimeric PilQ pore complex). Pilus-associated mutations may be important mechanisms of antibiotic escape for commensal species as there is some evidence that they do not require type IV pili for host-cell attachment (83); however, they may have a high fitness cost in *Neisseria* pathogens which do require functional pili (84) and thus are not likely candidates for long-term persistence in *N. gonorrhoeae or N. meningitidis* populations.

When investigating the haplotypes of each evolved strain, variants contributing to elevated MICs became more apparent in some cases (Table S4). For azithromycin selection, *N. canis* evolved MtrR mutations in all evolved replicates; however, only the addition of MtrD E823K or RplV tandem duplications was sufficient to elevate MICs > 16 µg/mL (the base MIC after one MtrR mutation was acquired). For *N. subflava*, duplications in RplV increased MICs more than those in RpmH (256 µg/mL vs 96 µg/mL, respectively). For penicillin-based selection in *N. cinerea*, the PenA A549E mutation had the largest impact on elevated MICs compared to the other PenA mutations that emerged (V548E and F518S). For *N. cinerea* as well, the RpoB P591S alone raised MICs from 2 µg/mL to 6 µg/mL. For *N. canis*, though all strains evolved resistance through acquisition of PenA and MtrD mutations, the haplotype with the highest MIC was PenA V574E and MtrD V139G (12 µg/mL compared to ≤2 µg/mL). Finally, in reviewing ancestral strains with above breakpoint MICs (Table 1), some mutations that were evolved in this study or identified previously were present that may further support their involvement in reduced susceptibility. For example, in the ancestral *N. cinerea*, we find MtrR A39T and G172D. However, in the ancestral *N. subflava* strains, we do not find described resistance mutations, suggesting there are more contributors to be identified.

**Fig 6:**
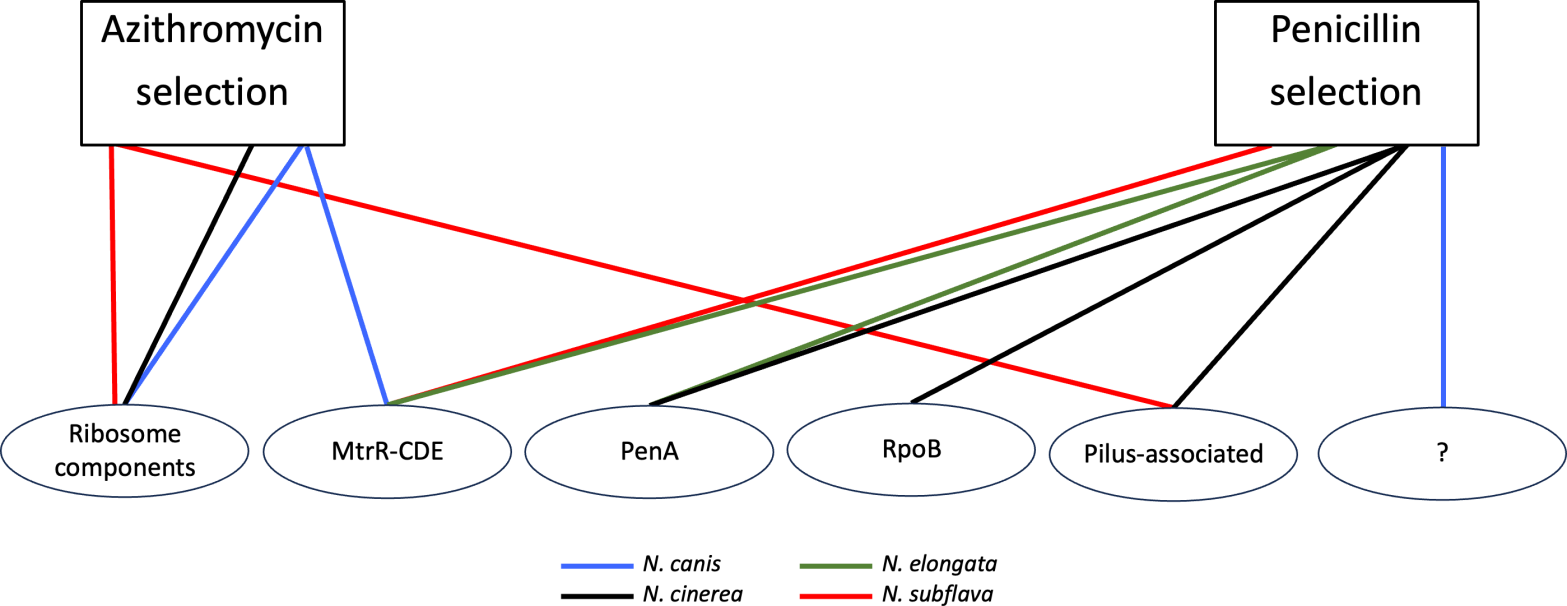
Preliminary map of the paths to resistance emergence across commensal members of the *Neisseria* genus. For azithromycin selection, all species with evolved resistance converged on described *Neisseria* resistance mutations within ribosome components, the *mtrRCDE* efflux pump system, or *pilQ*. For penicillin resistance: *N.cinerea*, *N. elongata*, and *N. subflava* also converged on described *Neisseria* resistance mutations within the *mtrRCDE* efflux pump system, *rpoB*, *pilM*, or *penA. N. canis* experimental replicates evolving penicillin resistance acquired as-of-yet undescribed resistance mutations.

The aforementioned ribosomal, MtrRCDE, PenA, RpoB, and pilus-associated mechanisms seem to be the likely contributors to the emergence of reduced susceptibly in all of the *Neisseria* commensals investigated in this study for both penicillin and azithromycin-based selection (Fig. 6). Therefore, despite 2/2 *N. canis* replicates evolving low-level penicillin resistance with as-of-yet unexplained genetic bases, with 19/21 cases of *Neisseria* evolution converging on known resistance modalities described in other *Neisseria* species (25, 68, 82), we must accept a constrained range of adaptive solutions to antibiotic selection within the genus at this point. The remaining questions do exist, however. For example, here, we primarily investigate coding-domain regions; thus, important mutations in intergenic regions such as promoters could have been missed. However, we specifically searched for mutations within the *mtrR-CDE* and *macAB* promoter regions because known resistance-conferring mutations within the regions have been identified previously (29, 60, 85) yet we found none within this study. We also acknowledge that our small sample of strains and experimental replicates, and subsampling of each evolved lineage by selecting a single colony, may have limited the pool of potential resistance mechanisms uncovered. For example, some mechanisms may be less frequently observed due to high fitness costs, necessitating the evolution of compensatory mutations. These types of mutations may, therefore, be missed in small-scale experimental studies. Finally, evolution does not occur in controlled laboratory environments, so what is the role of intergenus gene exchange in *Neisseria* resistance emergence? For example, the β-lactamase-containing plasmid p*bla* present within the gonococcal and meningococcal populations was acquired from the pathogenic *Haemophilus ducreyi* (86, 87). Therefore, are there other clinically relevant resistance mechanisms available to the *Neisseria* that could be acquired from other inhabitants of the nasopharyngeal mucosa, urogenital tract, or rectum [see references (45) for additional discussion]? In summary, our current results highlight conserved paths to resistance within the *Neisseria* genus, though continued tosses of the evolutionary dice across different drug classes (e.g., tetracyclines, quinolones, etc.) and strain combinations may ultimately paint a different picture which will be the focus of future experiential learning opportunities within undergraduate classrooms at RIT.

## MATERIALS AND METHODS

### Bacterial strains and culturing

Stocks of *Neisseria* were obtained from the Centers for Disease Control and Prevention and Food and Drug Association’s Antibiotic Resistance (AR) Isolate Bank “*Neisseria* species MALDI-TOF Verification panel.” Evolved strains included: AR-0944 (*N. cinerea*), AR-0945 (*N. elongata*), AR-0948 (*N. canis*), AR-0953 (*N. subflava*), and AR-0957 (*N. subflava*). Bacteria were cultivated for all subsequent protocols on GC agar base (Becton Dickinson Co., Franklin Lakes, NJ, USA) media plates containing 1% Kelloggs solution (GCP-K plates) for 18–24 hours at 37°C in a 5% CO_2_ atmosphere. Bacterial stocks were stored in trypticase soy broth (TSB) containing 20% glycerol at −80°C.

### Experimental evolution, MIC testing, and growth curves

MICs were measured by Etest strips (bioMérieux, Durham, NC) on GCB-K plates according to the manufacturer’s specifications. In brief, cells from overnight plates were suspended in TSB to a 0.5 McFarland standard, and 200 µL of the suspension subsequently inoculated onto GCB-K plates. Etest strips were incubated on these plates for 18–24 hours at 37°C in a 5% CO_2_ incubator. MICs were subsequently determined by reading the lowest concentration that inhibited the growth of bacterial lawns. Etests have been demonstrated to report equivalent MICs to the agar-dilution method for *Neisseria* (88).

For each of the four *Neisseria sp.* used in the study, four replicates were passaged on GCB-K plates containing a selective gradient of either penicillin or azithromycin. Selective gradients were created using Etest strips as described above and previously (5). Cells to be passaged were collected from the entire ZOI and a 1 cm region in the bacterial lawn surrounding the ZOI (Fig. 1) from the prior day’s plate, suspended in TSB to a ~0.5 McFarland standard, and 200 µL spread onto a new GCB-K plate containing a fresh Etest strip. MICs were recorded each day of the experiment. Strains were exposed to azithromycin and penicillin for 20 days. Controls for each species were passaged on GCB-K plates as described above; however, they did not contain any antibiotic.

Doubling time was assessed for each strain used within the study. In brief, overnight cultures on GCB-K media were inoculated into GCP broth supplemented with 1% Kelloggs solution to an OD_600_ of ~0.1 (*n* = 4 replicate suspensions per species). OD_600_ readings were taken using a Genesys 150 spectrophotometer (Thermo Scientific, Waltham, MA, USA) with measurements taken every 30 minutes for 24 hours with a 30-second shake at 180 cpm prior to reading. The BioTek Gen5 v.3.05 software was used for data interpretation and export.

### Genomic sequencing and comparative genomics

DNA was isolated from cells using the PureLink Genomic DNA Mini kit (Thermo Fisher Corp., Waltham, MA, USA), following lysis in TE buffer [10 mM Tris (pH 8.0) and 10 mM EDTA] with 0.5 mg/mL lysozyme and 3 mg/mL proteinase K (Sigma-Aldrich Corp., St. Louis, MO, USA). The resultant genomic DNA was treated with RNase A and prepared for sequencing using the Nextera XT kit (Illumina Corp., San Diego, CA, USA). Libraries were uniquely dual-indexed and pooled and sequenced on the Illumina MiSeq platform at the Rochester Institute of Technology Genomics Core using V3 600 cycle cartridges (2 × 300 bp). The sequencing quality of each paired-end read library was assessed using FastQC v0.11.9 (89). Trimmomatic v0.39 (90) was used to trim adapter sequences and remove bases with Phred quality score <15 over a 4 bp sliding window. Reads <36 bp long, or those missing a mate, were also removed from subsequent analysis. Draft assemblies had been previously published for all strains (54), except for *N. cinerea* AR-0944. This assembly was constructed using SPAdes v.3.14.1 (91), and all assemblies were annotated with Bakta v.1.8.1 (92). Assembly quality was assessed using QUAST (http://cab.cc.spbu.ru/quast/). Trimmed reads were mapped back to draft assemblies using Bowtie2 v.2.2.4 (93) using the “end-to-end” and “very-sensitive” options, and Pilon v.1.16 (94) was used to call variant sites. Data analysis and visualizations were conducted in R (95).

## Data Availability

Read libraries generated within this study can be accessed through NCBI’s Sequence Read Archive as a part of the BioProject PRJNA1018855. The AR-0944 reference assembly is also available on NCBI (accession: JAXUDV000000000). All scripts and datasetsdata sets are available on: https://github.com/wadsworthlab.

## SUPPLEMENTAL MATERIAL

Figure S1 spectrum.03507-23-s0001.tiff Growth curves of *Neisseria* commensals in GCP-K liquid media at 37{degree sign}C.

Figure S2 spectrum.03507-23-s0002.tiff Ancestral azithromycin MICs started significantly higher across species compared to penicillin MICs (P < 0.001).

Tables S2 and S3 spectrum.03507-23-s0003.xlsx ANOVA results.

Table S1 spectrum.03507-23-s0004.xlsx MICs for single colony picks of evolved lineages.

Table S4 spectrum.03507-23-s0005.xlsx Haplotypes of candidate resistance-contributing loci for strains evolving resistance in the study.

ASM does not own the copyrights to Supplemental Material that may be linked to, or accessed through, an article. The authors have granted ASM a non-exclusive, world-wide license to publish the Supplemental Material files. Please contact the corresponding author directly for reuse.
