# Supplementary figures and images for: Rolling the evolutionary dice: Neisseria commensals as proxies for elucidating the underpinnings of antibiotic resistance mechanisms and evolution in human pathogens
Source: Microbiol Spectr. 2024 Jan 5;12(2):e03507-23. doi: 10.1128/spectrum.03507-23 (PMC10871548; doi:10.1128/spectrum.03507-23)

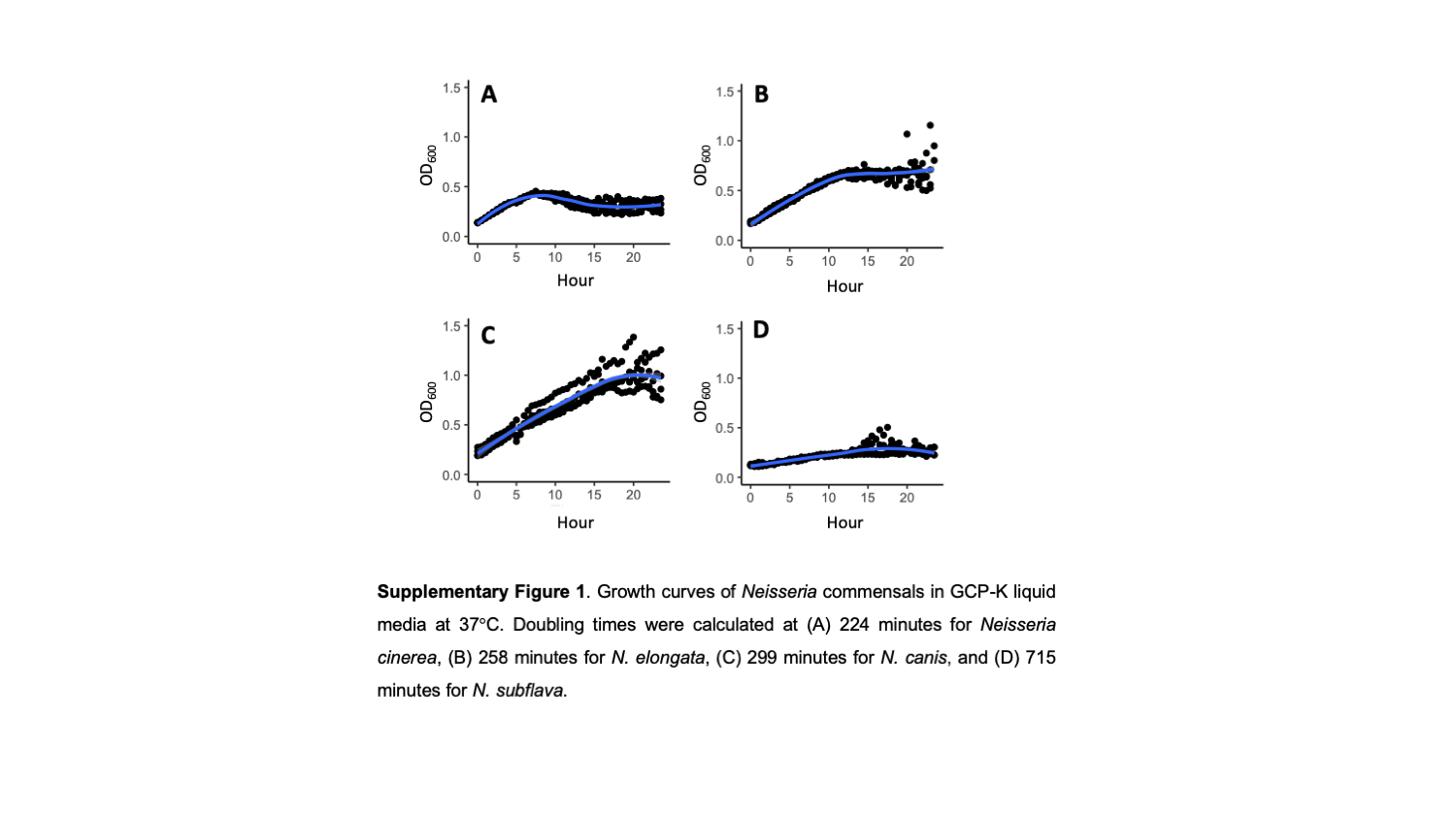

Supplement: Figure S1 — Growth curves of Neisseria commensals in GCP-K liquid media at 37{degree sign}C. [file spectrum.03507-23-s0001.tiff]

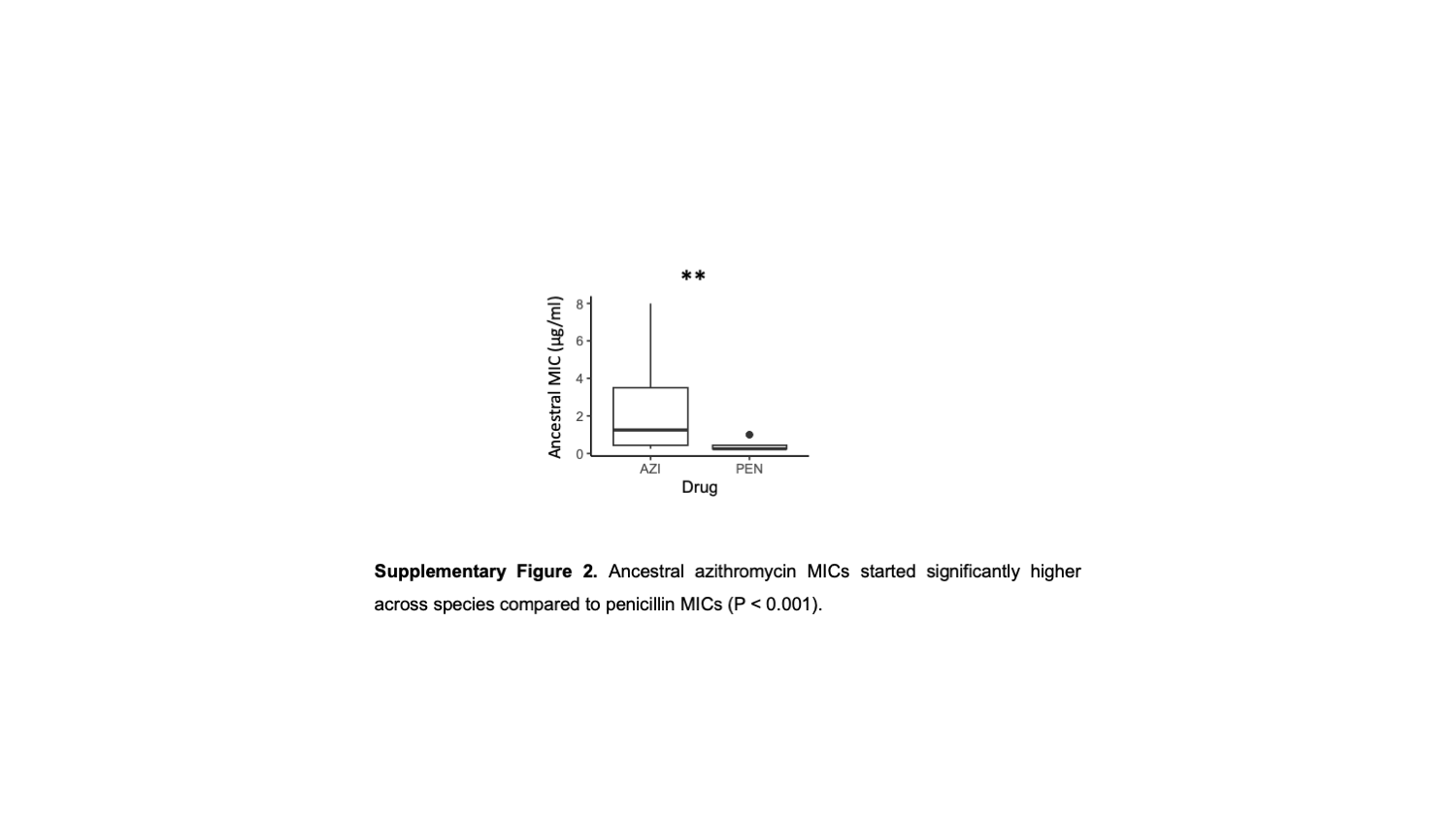

Supplement: Figure S2 — Ancestral azithromycin MICs started significantly higher across species compared to penicillin MICs (P < 0.001). [file spectrum.03507-23-s0002.tiff]
